# Serum WISP-1 and CTRP1 Levels in Gestational Diabetes Mellitus: Associations with HOMA-IR and Glycemic Status

**DOI:** 10.3390/medicina62101941

**Published:** 2026-10-08

**Authors:** Oznur Dundar Akin, Naile Fevziye Misirlioglu, Mete Hakan Karalok, Hafize Uzun

**Affiliations:** 1 Department of Obstetrics and Gynecology, Faculty of Medicine, Istanbul Atlas University, Istanbul 34403, Türkiye; hakan.karalok@atlas.edu.tr; 2 Department of Biochemistry, Faculty of Medicine, İstanbul Nişantaşı University, Istanbul 34398, Türkiye; nailemisirlioglu@gmail.com; 3 Department of Biochemistry, Faculty of Medicine, Istanbul Atlas University, Istanbul 34403, Türkiye; hafize.uzun@atlas.edu.tr

**Keywords:** gestational diabetes mellitus, WISP-1, CCN4, CTRP1, insulin resistance, HOMA-IR, adipokines

## Abstract

*Background and Objectives*: Gestational diabetes mellitus (GDM) is characterized by insulin resistance and metabolic dysregulation, in which adipokines may be involved. We evaluated serum Wnt1-inducible signaling pathway protein-1 (WISP-1/CCN4) and C1q/TNF-related protein-1 (CTRP1) in women with GDM and normal glucose tolerance and assessed their relationships with glucose–insulin homeostasis and cross-sectional discriminatory ability. *Materials and Methods*: This exploratory cross-sectional biomarker study with prospective obstetric follow-up included 100 pregnant women at 24–28 weeks of gestation (50 GDM and 50 controls). WISP-1, CTRP1, HOMA-IR, and GDM status were assessed during the same gestational period; therefore, the principal biomarker analyses were cross-sectional. GDM was diagnosed using the one-step 75-g oral glucose tolerance test criteria recommended by the American Diabetes Association Standards of Care 2026. WISP-1 and CTRP1 were measured by ELISA. Group comparisons, correlation analyses, ROC analyses, nested logistic regression, and bootstrap internal validation were performed. *Results*: WISP-1 was higher in GDM than in controls (422.80 [387.10–471.85] vs. 315.00 [288.30–351.55] pg/mL; *p* < 0.001), as was CTRP1 (34.13 ± 6.74 vs. 26.50 ± 5.65 ng/mL; *p* < 0.001). WISP-1 showed a higher apparent AUC than CTRP1 in the present cohort (0.908 vs. 0.802; DeLong *p* = 0.034). In the final model adjusted for maternal age and BMI, both WISP-1 (adjusted OR per 1-SD increase 7.53, 95% CI 2.95–19.21; *p* < 0.001) and CTRP1 (3.61, 95% CI 1.45–8.99; *p* = 0.006) remained associated with GDM status after adjustment for these covariates. Within GDM, the age- and BMI-adjusted association between WISP-1 and HOMA-IR remained significant (partial ρ = 0.574; *p* < 0.001), whereas CTRP1 was not associated with HOMA-IR. *Conclusions*: WISP-1 and CTRP1 were elevated in GDM, with WISP-1 showing a stronger association with HOMA-IR and a higher internally derived AUC in this cohort. These findings support further investigation of WISP-1 and CTRP1 as exploratory metabolic biomarkers associated with GDM but do not establish diagnostic or predictive utility. Independent external validation is required before any potential clinical application.

## 1. Introduction

Gestational diabetes mellitus (GDM) is one of the most common metabolic complications of pregnancy and is characterized by hyperglycemia first recognized during gestation in women without pre-existing diabetes. Its clinical importance extends beyond maternal glucose intolerance, as GDM is associated with an increased risk of adverse pregnancy outcomes, including preeclampsia, cesarean delivery, fetal overgrowth, neonatal adiposity, and birth-related complications [1]. Moreover, the consequences of GDM may persist beyond pregnancy. Long-term follow-up studies have demonstrated an increased risk of subsequent disturbances in maternal glucose metabolism and greater adiposity in the offspring, emphasizing that GDM represents not only a transient pregnancy-related disorder but also an early marker of future metabolic risk [2].

Pregnancy is physiologically accompanied by a progressive reduction in insulin sensitivity, particularly during the second and third trimesters, which facilitates nutrient availability for the growing fetus. Under normal conditions, pancreatic β-cells compensate for this pregnancy-induced insulin resistance by increasing insulin secretion. GDM develops when this adaptive response becomes insufficient to maintain normoglycemia. However, contemporary evidence indicates that the pathophysiology of GDM extends beyond the balance between insulin resistance and β-cell function and involves a complex interaction among adipose tissue dysfunction, placental signaling, chronic low-grade inflammation, and altered secretion of metabolically active adipokines [3]. Adipose tissue is now recognized as an active endocrine and immunometabolic organ, and changes in the maternal adipokine profile may contribute to the heterogeneous metabolic phenotype observed in GDM.

Wnt1-inducible signaling pathway protein 1 (WISP-1), also known as CCN4, is a secreted matricellular protein of the CCN family that has emerged as an adipokine linking adipose tissue dysfunction, inflammation, and impaired insulin action. WISP-1 expression in adipose tissue is associated with obesity and markers of insulin resistance, and experimental studies have demonstrated that WISP-1 may interfere with insulin signaling in skeletal muscle and hepatocytes [4,5]. These observations have stimulated interest in its potential role in pregnancy-related metabolic dysfunction. In women with GDM, Ersoy et al. reported higher circulating WISP-1 concentrations than in women with normal glucose tolerance, with positive associations with body mass index (BMI), fasting glucose, fasting insulin, and homeostatic model assessment of insulin resistance (HOMA-IR) [6]. Subsequent evidence also linked circulating WISP-1 with the coexistence of maternal overweight/obesity and GDM, supporting the hypothesis that WISP-1 may reflect the interaction between adiposity and dysglycemia during pregnancy [7].

C1q/TNF-related protein 1 (CTRP1), a member of the C1q/TNF-related protein family, represents another adipose tissue-derived signaling molecule implicated in glucose and lipid metabolism. In contrast to the predominantly adverse metabolic associations reported for WISP-1, the biological role of CTRP1 appears to be more complex and context dependent. Circulating CTRP1 has been associated with obesity, type 2 diabetes, insulin resistance, and inflammatory markers, whereas experimental studies have also suggested potentially compensatory or insulin-sensitizing effects [8]. Data regarding CTRP1 during pregnancy remain limited. In a cohort of women with GDM and normal glucose tolerance, Deischinger et al. found no significant overall difference in CTRP1 concentrations between the two groups; however, women with an insulin-resistant GDM phenotype had lower CTRP1 concentrations than those with a predominantly insulin-secretory defect and women with normal glucose tolerance. CTRP1 was also associated with indices of insulin sensitivity and glycemic regulation [9]. These findings suggest that circulating CTRP1 may provide metabolic information that is not captured by GDM diagnosis alone.

Importantly, WISP-1 and CTRP1 may represent distinct but interconnected components of adipose tissue signaling. A recent study in non-diabetic adults demonstrated a significant relationship between circulating WISP-1 and CTRP1 and suggested that these adipokines may have partially opposing or compensatory relationships with insulin sensitivity [10]. Nevertheless, their concurrent behavior in GDM and their relationships with conventional measures of insulin resistance and glycemic control remain insufficiently characterized. Previous pregnancy studies have largely evaluated these molecules individually and have predominantly focused on cross-sectional metabolic associations at the time of GDM diagnosis.

Therefore, the present study aimed to compare serum WISP-1 (CCN4) and CTRP1 concentrations between women with GDM and pregnant women with normal glucose tolerance and to evaluate their associations with insulin resistance and glycemic parameters. We also assessed the discriminatory performance of each adipokine and their combined contribution beyond maternal age and BMI. We hypothesized that concurrent assessment of these two metabolically relevant adipokines might help characterize their respective associations with the metabolic features of GDM beyond conventional glucose-based measures.

## 2. Materials and Methods

### 2.1. Ethical Considerations

The study protocol was reviewed and approved by the Istanbul Atlas University Clinical Research Ethics Committee on 16 June 2026 (Decision No. 2026/07-03; official document No. E-22686390-050.99-102075). The Ethics Committee determined that the study was ethically appropriate. All participants were informed about the purpose and procedures of the study, and written informed consent was obtained before enrollment. Participant enrollment commenced following ethics committee approval and continued prospectively until the prespecified sample size was achieved. The study was conducted in accordance with the principles of the Declaration of Helsinki.

### 2.2. Study Design and Participants

This single-center exploratory cross-sectional biomarker study with prospective obstetric follow-up until delivery was conducted at the Department of Obstetrics and Gynecology, Istanbul Atlas University Hospital, Istanbul, Türkiye. Participant recruitment was initiated after approval of the study protocol by the institutional ethics committee. Pregnant women undergoing routine screening for gestational diabetes mellitus (GDM) at 24–28 weeks of gestation were recruited and classified according to the results of the 75-g oral glucose tolerance test (OGTT). Recruitment continued until the prespecified sample size of 50 women with GDM and 50 women with normal glucose tolerance was reached. Women with normal OGTT results recruited during the same study period constituted the control group. The study therefore used a balanced sampling design and was not intended to reflect the prevalence of GDM in the routine screening population. Complete screening-log data, including the total numbers of women screened, eligible, excluded, or declining participation, were not systematically recorded. Because WISP-1, CTRP1, HOMA-IR, and GDM status were assessed during the same 24–28-week gestational period, the principal biomarker analyses were cross-sectional in nature. Participants were subsequently followed until delivery for prespecified maternal and perinatal outcomes.

A total of 100 pregnant women were included, comprising 50 women with GDM and 50 women with normal glucose tolerance who served as controls. All participants underwent a 75-g OGTT after an overnight fast. GDM was diagnosed according to the one-step 75-g OGTT criteria recommended by the American Diabetes Association (ADA) Standards of Care 2026 [11]. The diagnosis was established when at least one of the following plasma glucose thresholds was met or exceeded: fasting glucose ≥ 92 mg/dL, 1-h glucose ≥ 180 mg/dL, or 2-h glucose ≥ 153 mg/dL. Participants with glucose concentrations below all three diagnostic thresholds were classified as having normal glucose tolerance.

Eligible participants were women aged 18–40 years with a singleton pregnancy who underwent routine GDM screening at 24–28 weeks of gestation. Exclusion criteria were pre-existing type 1 or type 2 diabetes mellitus, multiple pregnancy, chronic systemic, metabolic, inflammatory, or autoimmune disease, active infection, renal or hepatic dysfunction, fetal anomaly, and use of medications that could substantially affect glucose metabolism or adipokine concentrations, including systemic corticosteroids. These criteria are consistent with the approved study protocol.

### 2.3. Clinical and Biochemical Assessment

Maternal demographic and clinical characteristics, including maternal age, gestational age, gravidity, parity, body weight, height, and BMI, were recorded at enrollment. Routine laboratory measurements obtained during antenatal care included fasting plasma glucose, 1-h and 2-h OGTT glucose concentrations, glycated hemoglobin (HbA1c), fasting insulin, C-reactive protein (CRP), and routine biochemical parameters.

BMI was calculated from body weight and height measured at the 24–28-week study visit and therefore represents gestational BMI rather than pre-pregnancy BMI.

Insulin resistance was estimated using the homeostatic model assessment of insulin resistance (HOMA-IR), which was calculated according to the following formula:

HOMA-IR = [fasting insulin (µIU/mL) × fasting glucose (mg/dL)]/405.

The protocol defined the relationships of WISP-1 and CTRP1 with fasting glucose, HbA1c, fasting insulin, HOMA-IR, CRP, and OGTT glucose concentrations as metabolic outcomes of interest.

### 2.4. Blood Sampling and Serum Preparation

Venous blood samples were obtained during routine antenatal assessment and GDM screening at 24–28 weeks of gestation after an overnight fast of approximately 8–10 h. Samples were collected in the morning between 08:00 and 10:00. No additional venipuncture was performed specifically for the study; residual serum remaining after completion of routine laboratory analyses was used for measurement of the study biomarkers.

Serum samples were separated by centrifugation at approximately 2376× *g* for 5 min using an Eppendorf 5424/5425 microcentrifuge equipped with an FA-45-24-11 rotor. Serum aliquots were transferred into coded Eppendorf tubes and stored at −80 °C until analysis. WISP-1 and CTRP1 measurements were performed after completion of sample collection to minimize analytical variability.

### 2.5. Measurement of Serum WISP-1/CCN4

Serum Wnt1-inducible signaling pathway protein 1 (WISP-1/CCN4) concentrations were determined using a commercially available human sandwich enzyme-linked immunosorbent assay (ELISA) kit (FineTest^®^, Human WISP1/CCN4 ELISA Kit, catalogue no. EH0330, revision V4.0; Wuhan Fine Biotech Co., Ltd., Wuhan, Hubei, China) according to the manufacturer’s instructions. The assay has a measurement range of 31.25–2000 pg/mL and a reported analytical sensitivity of 18.75 pg/mL.

Briefly, WISP-1/CCN4 in serum was captured by an antibody pre-coated onto the microplate and detected using a biotin-conjugated anti-WISP-1/CCN4 antibody followed by horseradish peroxidase–streptavidin. Following substrate development and termination of the enzymatic reaction, optical density was measured at 450 nm, and serum concentrations were calculated from the corresponding standard curve.

According to the manufacturer, the intra-assay coefficients of variation (CVs) across low-, medium-, and high-concentration samples ranged from 4.69% to 5.70%, whereas the inter-assay CVs ranged from 5.73% to 6.02%. The reported mean recovery in serum was 98%.

### 2.6. Measurement of Serum CTRP1

Serum C1q/TNF-related protein 1 (CTRP1; C1QTNF1) concentrations were measured using a human sandwich ELISA kit (FineTest^®^, Human C1QTNF1 ELISA Kit, catalogue no. EH1624, revision V4.0; Wuhan Fine Biotech Co., Ltd., Wuhan, Hubei, China) according to the manufacturer’s protocol. The measurement range of the assay was 3.125–200 ng/mL, with a reported analytical sensitivity of 1.875 ng/mL.

The assay was based on an antibody-sandwich principle using a C1QTNF1-specific capture antibody and a biotinylated detection antibody. After addition of HRP–streptavidin and TMB substrate, absorbance was measured at 450 nm, and CTRP1 concentrations were derived from the standard curve.

The manufacturer-reported intra-assay CVs ranged from 5.23% to 5.91%, and inter-assay CVs ranged from 4.82% to 6.32%. Mean recovery in serum was 94%.

All WISP-1 and CTRP1 measurements were performed in the Istanbul Atlas University Biochemistry Laboratory using standardized procedures. Samples were analyzed using the same assay platform and kit lots, and measurements were performed in duplicate as specified in the study protocol.

### 2.7. Exploratory Maternal and Perinatal Outcomes

Following baseline assessment at 24–28 weeks of gestation, participants were prospectively followed through their routine antenatal visits until delivery. No additional study-specific visits were scheduled.

For women diagnosed with GDM, subsequent glycemic management was documented during follow-up as part of the approved protocol. Maternal and perinatal outcomes, including preeclampsia, gestational age at delivery, mode of delivery, neonatal birth weight, macrosomia, Apgar scores, and neonatal intensive care unit requirement, were also recorded for all participants during follow-up. Complete maternal and perinatal outcome data were available for all 100 participants (50 women with GDM and 50 women with normal glucose tolerance), with no loss to follow-up for these variables. Pregnancy outcomes were prespecified in the study protocol as secondary clinical outcomes and were analyzed descriptively and exploratorily because the study was not specifically powered to detect between-group differences in individual maternal or neonatal outcomes. Given the low number of events for several outcomes, these analyses were not used to support definitive inferential conclusions, particularly when *p* values were close to the conventional significance threshold.

### 2.8. Sample Size Calculation

The target sample size was prespecified before participant recruitment for the primary comparison of circulating adipokine concentrations between women with GDM and women with normal glucose tolerance. Sample-size planning was based on a two-sided comparison of two independent groups with equal allocation, an α level of 0.05, and 80% statistical power. With 50 participants per group, the planned sample size of 100 participants provides 80% power to detect a standardized between-group difference of approximately Cohen’s d = 0.57. The study was not specifically powered for multivariable logistic regression, ROC analyses, or individual maternal and neonatal outcomes; these analyses were therefore considered exploratory.

### 2.9. Statistical Analysis

Statistical analyses were performed using Python 3.13.5 (SciPy 1.17.0, statsmodels 0.14.6, and scikit-learn 1.8.0). Continuous variables were assessed for distributional characteristics using visual inspection and the Shapiro–Wilk test. Normally distributed continuous variables are presented as the mean ± standard deviation (SD), whereas non-normally distributed variables are presented as the median [interquartile range]. Categorical variables are expressed as a number and percentage.

Maternal age and BMI were selected a priori as clinically relevant covariates because of their established relationships with GDM risk, insulin resistance, and adipokine concentrations. Given the sample size, the primary multivariable model was deliberately restricted to a limited number of prespecified covariates to reduce the risk of model overfitting.

Comparisons between the GDM and control groups were performed using the independent-samples Student’s *t* test for normally distributed continuous variables and the Mann–Whitney U test for non-normally distributed variables. Categorical variables were compared using the chi-square test or Fisher exact test, as appropriate. Between-group differences in WISP-1 and CTRP1 were additionally quantified using Hedges’ g with bootstrap confidence intervals.

Associations of serum WISP-1 and CTRP1 concentrations with fasting glucose, HbA1c, fasting insulin, HOMA-IR, CRP, OGTT glucose concentrations, BMI, and lipid parameters were evaluated using Pearson correlation coefficients (r) for approximately normally distributed variable pairs or Spearman rank correlation coefficients (ρ) for non-normally distributed pairs. The prespecified primary hypotheses concerned the between-group differences in WISP-1 and CTRP1 concentrations, whereas the correlation analyses were considered secondary and exploratory. To address multiple testing, the Benjamini–Hochberg false-discovery-rate procedure was applied across all 24 biomarker–clinical correlations as a single family, with q < 0.05 considered statistically significant. Within the GDM group, partial Spearman correlations adjusted for maternal age and BMI were additionally calculated to assess the associations of WISP-1 and CTRP1 with HOMA-IR after adjustment for these covariates.

Receiver operating characteristic (ROC) curve analysis was performed to evaluate the ability of WISP-1 and CTRP1 to discriminate women with GDM from those with normal glucose tolerance. Areas under the ROC curves (AUCs) with 95% confidence intervals were calculated, and exploratory, internally derived cut-off values were identified using the Youden index, with corresponding sensitivity and specificity determined. A combined WISP-1 + CTRP1 model was generated from binary logistic regression-predicted probabilities. Correlated AUCs were compared using the DeLong test. AUC confidence intervals were estimated using nonparametric bootstrap resampling (3000 repetitions). The individual WISP-1 and CTRP1 ROC AUCs were treated as apparent, in-sample estimates and were not subjected to optimism correction; bootstrap optimism correction was only applied to the final multivariable model.

To evaluate the incremental contribution of the adipokines beyond basic maternal characteristics, nested binary logistic regression models were constructed sequentially as follows: age + BMI; age + BMI + WISP-1; age + BMI + CTRP1; and age + BMI + WISP-1 + CTRP1. Model performance was assessed using AUC, Akaike information criterion (AIC), Nagelkerke R^2^, and likelihood-ratio tests for appropriate nested model comparisons. Because WISP-1 and CTRP1 were measured on substantially different numerical scales, both biomarkers were standardized before the final multivariable model, and adjusted odds ratios are reported per 1-SD increase in biomarker concentration with 95% confidence intervals.

Internal validation of the final age + BMI + WISP-1 + CTRP1 model was performed using bootstrap resampling (1000 repetitions). Bootstrap optimism was estimated as the mean difference between model performance within each bootstrap sample and the performance of the corresponding bootstrap-fitted model when applied to the original dataset. The optimism-corrected AUC was obtained by subtracting the estimated optimism from the apparent AUC. Calibration of the final multivariable model including maternal age, BMI, WISP-1, and CTRP1 was assessed using the calibration intercept, calibration slope, and Brier score. Optimism-corrected calibration estimates were obtained using the same 1000-repetition bootstrap internal-validation procedure. Complete biomarker, clinical, and laboratory data required for the prespecified analyses were available for all 100 participants. No missing values were present in the variables included in the primary analyses; therefore, no imputation or other missing-data handling procedure was required. Because gestational age at biomarker assessment could potentially influence metabolic and adipokine concentrations, an additional sensitivity analysis was performed by adding gestational age at the 24–28-week study visit to the final multivariable model.

All statistical tests were two-sided, and *p* < 0.05 was considered nominally significant. For the family of 24 exploratory biomarker–clinical correlations, FDR-adjusted q < 0.05 was considered statistically significant.

## 3. Results

### 3.1. Participant Characteristics

A total of 100 pregnant women were included, comprising 50 women with GDM and 50 women with normal glucose tolerance who served as controls. Age and gestational age at enrollment were similar between groups (30.26 ± 4.39 vs. 30.80 ± 4.81 years, *p* = 0.559; and 26.50 [26.00–27.00] vs. 27.00 [26.00–27.00] weeks, *p* = 0.589, respectively). BMI was higher in the GDM group (29.94 ± 3.85 vs. 26.27 ± 3.10 kg/m^2^, *p* < 0.001). Diastolic blood pressure was also higher in women with GDM (*p* = 0.003), whereas systolic blood pressure did not differ significantly. Fasting glucose, 1-h and 2-h OGTT glucose, HbA1c, fasting insulin, HOMA-IR, and CRP were all higher in women with GDM (all *p* < 0.001). LDL cholesterol and triglycerides were higher in the GDM group, whereas HDL cholesterol did not differ significantly between groups (Table 1).

### 3.2. Serum WISP-1 and CTRP1 Concentrations

Serum WISP-1 concentrations were higher in women with GDM than in controls (422.80 [387.10–471.85] vs. 315.00 [288.30–351.55] pg/mL, *p* < 0.001). Serum CTRP1 concentrations were also higher in the GDM group (34.13 ± 6.74 vs. 26.50 ± 5.65 ng/mL, *p* < 0.001) (Table 1). The between-group effect sizes were large for both adipokines: Hedges’ g was 1.89 for WISP-1 (95% bootstrap CI 1.44–2.50) and 1.22 for CTRP1 (95% bootstrap CI 0.83–1.70).

### 3.3. Correlations with Metabolic and Glycemic Parameters

Within the GDM group, WISP-1 was positively correlated with fasting insulin (ρ = 0.441, *p* = 0.001) and HOMA-IR (ρ = 0.492, *p* < 0.001). After Benjamini–Hochberg correction across all 24 exploratory correlations, both associations remained statistically significant (q = 0.016 and q = 0.0069, respectively). Other nominal associations of WISP-1 did not remain significant after FDR correction. No correlation involving CTRP1 remained statistically significant after correction for multiple testing. Correlations of WISP-1 and CTRP1 with metabolic and glycemic parameters within the GDM group (n = 50).

Within the GDM group, WISP-1 was positively correlated with fasting insulin (ρ = 0.441, *p* = 0.001) and HOMA-IR (ρ = 0.492, *p* < 0.001). Both associations remained statistically significant after Benjamini–Hochberg correction across all 24 exploratory correlations (q = 0.016 and q = 0.0069, respectively). Although nominal associations were observed between WISP-1 and several other metabolic variables, including an inverse correlation with 1-h OGTT glucose, none remained significant after FDR correction and these findings were not interpreted further. No correlation involving CTRP1 remained significant after FDR correction (Table 2).

### 3.4. Discriminatory and Multivariable Analyses

**Figure 1:**
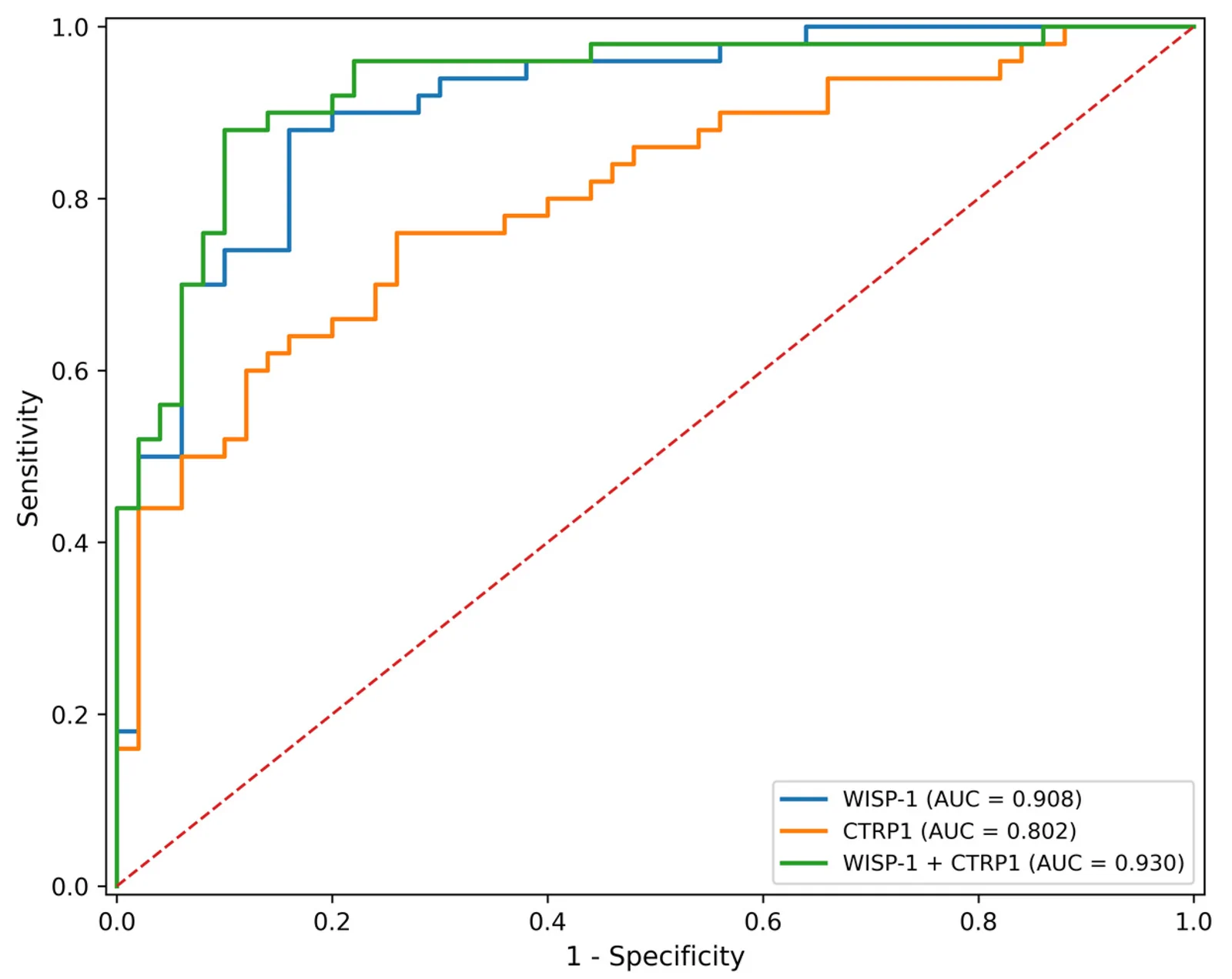
ROC curves of WISP-1, CTRP1, and the combined WISP-1 + CTRP1 model for discrimination of GDM. ROC curves of WISP-1, CTRP1, and the combined WISP-1 + CTRP1 model for discrimination of GDM. The red dotted diagonal line represents the reference line for no discriminatory ability (AUC = 0.50).

ROC analysis showed a higher apparent, in-sample AUC for WISP-1 than for CTRP1. WISP-1 yielded an apparent AUC of 0.908 (95% CI 0.847–0.962), with an exploratory, internally derived cut-off of 367.7 pg/mL (sensitivity 88%, specificity 84%). CTRP1 yielded an apparent AUC of 0.802 (95% CI 0.711–0.883), with an exploratory, internally derived cut-off of 30.71 ng/mL (sensitivity 76%, specificity 74%). These thresholds were derived within the present cohort and should not be interpreted as clinically established diagnostic cut-offs. The AUC of WISP-1 was significantly greater than that of CTRP1 by the DeLong test (*p* = 0.034). A model combining WISP-1 and CTRP1 yielded an AUC of 0.930 (95% CI 0.875–0.977), but did not significantly outperform WISP-1 alone (DeLong *p* = 0.190) (Figure 1).

The incremental contribution of the adipokines was assessed using nested logistic regression models. The age + BMI model yielded an AUC of 0.773, an AIC of 119.86, and a Nagelkerke R^2^ of 0.293. Adding WISP-1 increased the AUC to 0.926 and improved model fit (likelihood-ratio χ^2^ = 43.64, *p* < 0.001), whereas adding CTRP1 increased the AUC to 0.860 (χ^2^ = 23.35, *p* < 0.001). The full model including age, BMI, WISP-1, and CTRP1 showed the best overall fit (AUC 0.940, 95% CI 0.895–0.980; AIC 70.94; Nagelkerke R^2^ 0.720). Addition of CTRP1 to the age + BMI + WISP-1 model further improved model fit (χ^2^ = 9.28, *p* = 0.002). Bootstrap internal validation yielded an optimism-corrected AUC of 0.926 for the full model (Table 3).

In the final multivariable logistic regression model including maternal age, BMI, WISP-1, and CTRP1, higher concentrations of both adipokines remained associated with GDM status after adjustment for the included covariates. Per 1-SD increase, WISP-1 was associated with an adjusted OR of 7.53 (95% CI 2.95–19.21; *p* < 0.001), and CTRP1 was associated with an adjusted OR of 3.61 (95% CI 1.45–8.99; *p* = 0.006). BMI also remained associated with GDM status after multivariable adjustment (adjusted OR 1.26 per kg/m^2^, 95% CI 1.01–1.56; *p* = 0.037), whereas maternal age was not (adjusted OR 0.96 per year, 95% CI 0.83–1.11; *p* = 0.562). Full regression coefficients, standard errors, odds ratios, 95% confidence intervals, and *p* values for all nested models are presented in Table 4. The final model had an apparent AUC of 0.940. After internal validation using 1000 bootstrap resamples, the optimism-corrected AUC was 0.926. Bootstrap internal validation also yielded an optimism-corrected calibration intercept of −0.016, calibration slope of 0.845, and Brier score of 0.117. The calibration slope below 1 suggests modest model overfitting. In a sensitivity analysis additionally adjusted for gestational age at the study visit, the results were materially unchanged. Gestational age was not statistically associated with GDM status (adjusted OR 1.27 per week, 95% CI 0.66–2.46; *p* = 0.473), while WISP-1 (adjusted OR per 1-SD increase 7.79, 95% CI 3.00–20.24; *p* < 0.001) and CTRP1 (adjusted OR per 1-SD increase 3.52, 95% CI 1.42–8.74; *p* = 0.007) remained associated with GDM status. BMI also remained associated with GDM status (adjusted OR 1.27 per kg/m^2^, 95% CI 1.02–1.58; *p* = 0.032). The apparent AUC of the sensitivity model was 0.940.

GDM status was coded as 1 and normal glucose tolerance as 0. WISP-1 and CTRP1 were standardized before regression; therefore, their ORs represent the change in odds associated with a 1-SD increase (79.14 pg/mL for WISP-1 and 7.28 ng/mL for CTRP1). Maternal age and BMI were entered in their original units. Model 1 included maternal age and BMI; Model 2 additionally included WISP-1; and Model 3 additionally included CTRP1.

Within the GDM group, the association between WISP-1 and HOMA-IR remained significant after adjustment for maternal age and BMI (partial Spearman ρ = 0.574, *p* < 0.001). In contrast, CTRP1 was not associated with HOMA-IR after adjustment for the same covariates (ρ = 0.076, *p* = 0.598).

### 3.5. Obstetric and Perinatal Outcomes

Delivery occurred at an earlier gestational week in the GDM group than in controls (38.00 [37.00–39.00] vs. 39.00 [38.00–40.00] weeks, *p* < 0.001). Birth weight did not differ significantly between groups (*p* = 0.076). No statistically significant between-group differences were observed in preeclampsia (*p* = 0.715), cesarean delivery (*p* = 0.529), macrosomia (*p* = 0.056), NICU requirement (*p* = 0.056), or preterm birth (*p* = 0.242) (Table 1).

Supplementary Figure S1. Calibration of the final multivariable logistic regression model. The calibration plot shows the relationship between predicted probabilities of gestational diabetes mellitus (GDM) and observed GDM proportions for the final model including maternal age, BMI, WISP-1, and CTRP1. The diagonal line represents ideal calibration, and the calibration curve represents bootstrap optimism-corrected model performance based on 1000 resamples. The optimism-corrected calibration intercept was −0.016, the calibration slope was 0.845, and the Brier score was 0.117. The calibration slope below 1 indicates modest model overfitting.

## 4. Discussion

The principal findings of the present study were that circulating WISP-1/CCN4 and CTRP1 concentrations were significantly higher in women with GDM than in women with normal glucose tolerance. WISP-1 showed consistent positive associations with fasting insulin and HOMA-IR within the GDM group, whereas CTRP1 was not significantly associated with these measures after restricting to women with GDM. WISP-1 also showed a higher AUC than CTRP1 in the present cohort (0.908 vs. 0.802; DeLong *p* = 0.034). In multivariable analysis adjusted for maternal age and BMI, both biomarkers remained associated with GDM status after adjustment for these covariates. However, the relatively large adjusted OR for WISP-1 (7.53 per 1-SD increase; 95% CI 2.95–19.21) should be interpreted cautiously given the modest sample size and wide confidence interval.

These findings are broadly consistent with current concepts of GDM as a heterogeneous metabolic disorder involving altered glucose–insulin homeostasis, adipose tissue dysfunction, inflammatory signaling, and changes in adipokine secretion [12,13,14]. Previous pregnancy-specific studies have similarly reported higher circulating WISP-1 concentrations in women with GDM and associations with BMI, fasting insulin, HOMA-IR, glucose, and triglycerides [6,7]. In the present study, the associations of WISP-1 with fasting insulin and HOMA-IR remained significant within the GDM group after correction for multiple testing, suggesting that they were not solely attributable to differences between the GDM and control groups. Nevertheless, because HOMA-IR is derived from fasting glucose and insulin, these findings should not be interpreted as demonstrating a specific relationship between WISP-1 and insulin resistance independent of glycemia.

Experimental studies have suggested that WISP-1 may interfere with insulin signaling in skeletal muscle and hepatocytes and may participate in adipose tissue inflammatory processes [5]. Although these observations provide biological context for the present findings, our study did not directly assess insulin signaling, adipose tissue function, or inflammatory pathways. The observed clinical associations should therefore be regarded as hypothesis-generating and do not establish a causal role for WISP-1 in GDM pathogenesis.

The CTRP1 findings were less straightforward. Circulating CTRP1 concentrations were higher in women with GDM, but CTRP1 was not significantly associated with fasting insulin or HOMA-IR within the GDM group. Previous pregnancy data are inconsistent; Deischinger et al. reported no overall difference in CTRP1 between women with GDM and those with normal glucose tolerance, although concentrations differed across metabolic GDM subtypes [9]. A recent systematic review and meta-analysis also reported higher CTRP1 concentrations in adults with type 2 diabetes, with substantial between-study heterogeneity [14]. Differences in adiposity, metabolic phenotype, gestational timing, and assay methodology may contribute to these inconsistencies. Although experimental studies have suggested insulin-sensitizing and glucose-regulatory actions of CTRP1, the present data cannot determine whether its elevation in GDM represents compensation, metabolic dysregulation, or another underlying process.

WISP-1 and CTRP1 were positively correlated in the pooled study population, but this relationship was no longer statistically significant after stratification by GDM status. Mirr et al. previously reported a moderate positive correlation between the two adipokines [10]. In our cohort, attenuation of the correlation after stratification suggests that the pooled association may partly reflect between-group separation rather than direct biological coupling. Thus, their simultaneous associations with GDM indicate nonredundant statistical information within this dataset but do not establish biological complementarity or independence. Several weaker nominal correlations, including the inverse association between WISP-1 and 1-h OGTT glucose, did not survive FDR correction and should therefore be considered exploratory.

The ROC analyses showed a higher AUC for WISP-1 than for CTRP1 in the present cohort. However, the AUC estimates and corresponding cut-off values were derived and evaluated within the same balanced single-center sample and should therefore be considered exploratory. The findings do not establish clinical superiority of WISP-1 or support either biomarker as an alternative to OGTT-based diagnosis. Independent validation in larger and more heterogeneous populations using prespecified thresholds is required before any potential clinical application [15].

Maternal adiposity is an important potential confounder. Women with GDM had higher BMI than controls, although WISP-1 and CTRP1 remained associated with GDM status after adjustment for maternal age and BMI. The BMI used in the present analyses was measured at 24–28 weeks and therefore represents gestational rather than pre-pregnancy BMI. Because pre-pregnancy BMI and gestational weight gain were not systematically available, residual confounding related to pre-pregnancy adiposity, pregnancy-related weight gain, body fat distribution, dietary factors, and other metabolic characteristics cannot be excluded. More comprehensive assessment of maternal body composition should therefore be incorporated into future biomarker studies [16].

Women with GDM delivered approximately 0.9 weeks earlier than controls. Specific obstetric indications for delivery were not systematically captured, and most neonatal outcomes showed no clear between-group differences. Because the study was not powered for individual maternal or neonatal outcomes and several events were infrequent, these findings should be regarded as descriptive and exploratory rather than interpreted on the basis of borderline *p* values.

## 5. Strengths and Limitations

The main strengths of the study include simultaneous evaluation of WISP-1 and CTRP1 within the same pregnancy cohort, standardized sampling during the 24–28-week gestational window, assessment of fasting insulin and HOMA-IR, and prospective follow-up until delivery.

Several limitations should be acknowledged. The single-center design and modest sample size limit generalizability. Biomarkers and GDM status were assessed during the same gestational period; therefore, the principal biomarker analyses were cross-sectional and cannot establish prospective prediction or early diagnostic performance. HOMA-IR is an indirect surrogate rather than a gold-standard measure of insulin sensitivity. Pre-pregnancy BMI and gestational weight gain were unavailable, leaving potential residual confounding related to maternal adiposity. The study was also not specifically powered for multivariable regression, ROC analyses, or individual pregnancy outcomes. Finally, the balanced inclusion of 50 women with GDM and 50 controls, absence of a complete screening log, and lack of an external validation cohort may limit the generalizability of the internally derived AUC estimates and cut-off values to an unselected screening population.

## 6. Clinical Implications and Future Directions

The present findings support further investigation of WISP-1 and CTRP1 as exploratory metabolic biomarkers associated with GDM, but their incremental clinical value remains uncertain. This approach is consistent with recent biomarker studies emphasizing multimarker strategies that integrate adipokine, inflammatory, metabolic, and maternal clinical information rather than relying on a single circulating marker [14,17,18,19,20]. Future studies should use larger multicenter cohorts, longitudinal biomarker measurements preceding GDM ascertainment, direct measures of insulin sensitivity, detailed assessment of pre-pregnancy adiposity and gestational weight gain, and independent external validation.

## 7. Conclusions

Women with GDM had higher circulating WISP-1 and CTRP1 concentrations than women with normal glucose tolerance. WISP-1 showed a stronger association with HOMA-IR and related metabolic measures and a higher AUC for distinguishing women with GDM from those with normal glucose tolerance in the present cohort, whereas the relationship of CTRP1 with the metabolic phenotype appeared less directly linked to HOMA-IR within the GDM group. The associations of both adipokines with GDM status after adjustment for maternal age and BMI suggest that WISP-1 and CTRP1 may capture partly different statistical features within the present dataset; however, these findings do not establish distinct, independent, or complementary biological roles. WISP-1 appeared more closely associated with HOMA-IR and related glycemic–metabolic abnormalities, whereas the role of CTRP1 in GDM appeared more complex and potentially context-dependent. Because HOMA-IR is derived from fasting glucose and insulin, these findings should not be interpreted as demonstrating a specific association between WISP-1 and insulin resistance independent of glycemia. These findings support further investigation of both adipokines as exploratory metabolic biomarkers associated with GDM rather than as established diagnostic or predictive markers or replacements for OGTT-based testing. Larger, longitudinal, multicenter studies with biomarker assessment preceding GDM ascertainment and independent external validation are needed to clarify the biological relevance of WISP-1 and CTRP1 and to determine whether either biomarker, alone or in combination, provides reproducible and clinically meaningful information for metabolic phenotyping or future risk stratification in women at risk of GDM.

## Figures and Tables

**Table 1:** Clinical, metabolic, biomarker, and perinatal characteristics of the study groups.

Variable	GDM (n = 50)	Control (n = 50)	*p* Value
**Age (years)**	30.26 ± 4.39	30.80 ± 4.81	0.559
**Gestational week at enrollment**	26.50 [26.00–27.00]	27.00 [26.00–27.00]	0.589
**Gravida**	2.50 [2.00–4.00]	2.00 [2.00–3.00]	0.144
**Parity**	0.50 [0.00–1.00]	1.00 [0.00–1.00]	0.988
**BMI (kg/m^2^)**	29.94 ± 3.85	26.27 ± 3.10	<0.001
**Systolic blood pressure (mmHg)**	114.66 ± 10.77	111.56 ± 9.13	0.124
**Diastolic blood pressure (mmHg)**	73.90 ± 6.53	69.82 ± 6.84	0.003
**Fasting glucose (mg/dL)**	99.45 [93.62–108.40]	84.75 [80.53–87.95]	<0.001
**1-h OGTT glucose (mg/dL)**	184.66 ± 23.68	133.38 ± 17.86	<0.001
**2-h OGTT glucose (mg/dL)**	160.12 ± 25.91	111.09 ± 13.64	<0.001
**HbA1c (%)**	5.67 [5.52–5.93]	5.15 [5.02–5.39]	<0.001
**Fasting insulin (µIU/mL)**	15.80 [12.69–20.59]	8.82 [7.13–10.50]	<0.001
**HOMA-IR**	4.03 [3.15–5.15]	1.80 [1.54–2.21]	<0.001
**CRP (mg/L)**	4.80 [3.27–6.89]	2.74 [1.96–3.82]	<0.001
**Total cholesterol (mg/dL)**	238.53 ± 35.14	223.89 ± 41.31	0.059
**LDL cholesterol (mg/dL)**	141.80 ± 29.03	119.82 ± 30.75	<0.001
**HDL cholesterol (mg/dL)**	62.31 ± 12.04	62.84 ± 10.45	0.815
**Triglycerides (mg/dL)**	209 [171–245]	158 [140–195]	<0.001
**WISP-1/CCN4 (pg/mL)**	423 [387–472]	315 [288–352]	<0.001
**CTRP1/C1QTNF1 (ng/mL)**	34.13 ± 6.74	26.50 ± 5.65	<0.001
**Delivery week**	38.00 [37.00–39.00]	39.00 [38.00–40.00]	<0.001
**Birth weight (g)**	3349 ± 436	3193 ± 432	0.076
**Preeclampsia, n (%)**	5 (10.0%)	3 (6.0%)	0.715
**Cesarean delivery, n (%)**	19 (38.0%)	16 (32.0%)	0.529
**Macrosomia ≥ 4000 g, n (%)**	5 (10.0%)	0 (0.0%)	0.056
**NICU requirement, n (%)**	5 (10.0%)	0 (0.0%)	0.056
**Preterm birth < 37 weeks, n (%)**	3 (6.0%)	0 (0.0%)	0.242

Data are presented as mean ± SD, median [interquartile range], or n (%), as appropriate. Continuous variables were compared using the independent-samples Student’s *t* test or Mann–Whitney U test according to distribution; categorical variables were compared using chi-square or Fisher exact tests. **BMI**, body mass index; **CRP**, C-reactive protein; **GDM**, gestational diabetes mellitus; **HOMA-IR**, homeostatic model assessment of insulin resistance; **NICU**, neonatal intensive care unit; **OGTT**, oral glucose tolerance test.

**Table 2:** Exploratory correlations of WISP-1 and CTRP1 with metabolic parameters in women with GDM.

Variable	WISP-1 r/ρ	*p* Value	q Value	CTRP1 r/ρ	*p* Value	q Value
**Fasting glucose (mg/dL)**	r = 0.059	0.682	0.805	r = −0.068	0.637	0.805
**HbA1c (%)**	ρ = −0.149	0.302	0.724	ρ = −0.055	0.704	0.805
**Fasting insulin (µIU/mL)**	ρ = 0.441	0.001	**0.016**	ρ = 0.071	0.626	0.805
**HOMA-IR**	ρ = 0.492	<0.001	**0.0069**	ρ = 0.056	0.700	0.805
**CRP (mg/L)**	ρ = 0.240	0.093	0.320	ρ = 0.159	0.270	0.720
**1-h OGTT glucose (mg/dL)**	r = −0.297	0.036	0.157	r = −0.015	0.919	0.959
**2-h OGTT glucose (mg/dL)**	r = 0.189	0.189	0.567	r = 0.088	0.543	0.805
**BMI (kg/m^2^)**	r = 0.337	0.017	0.123	r = 0.096	0.509	0.805
**Total cholesterol (mg/dL)**	r = −0.292	0.039	0.157	r = 0.000	1.000	1.000
**LDL cholesterol (mg/dL)**	r = 0.126	0.383	0.805	r = −0.119	0.410	0.805
**HDL cholesterol (mg/dL)**	r = −0.113	0.436	0.805	r = 0.069	0.634	0.805
**Triglycerides (mg/dL)**	ρ = 0.327	0.020	0.123	ρ = 0.037	0.800	0.872

Pearson correlation coefficients (r) were used for approximately normally distributed variable pairs and Spearman rank coefficients (ρ) for non-normally distributed pairs. Reported *p* values are nominal, unadjusted two-sided values. Benjamini–Hochberg false-discovery-rate (FDR) correction was applied across all 24 exploratory biomarker–clinical correlations as a single family. An FDR-adjusted q < 0.05 was considered statistically significant. After correction, only the associations of WISP-1 with fasting insulin (q = 0.016) and HOMA-IR (q = 0.0069) remained statistically significant. Bold values indicate correlations that remained statistically significant after FDR correction. **BMI**, body mass index; **CRP**, C-reactive protein; **GDM**, gestational diabetes mellitus; **HOMA-IR**, homeostatic model assessment of insulin resistance; **OGTT**, oral glucose tolerance test.

**Table 3:** Incremental discriminatory performance of WISP-1 and CTRP1 for GDM.

Model	Apparent AUC (95% CI)	AIC	Nagelkerke R^2^
**Age + BMI**	0.773 (0.680–0.852)	119.86	0.293
**Age + BMI + WISP-1**	0.926 (0.875–0.971)	78.22	0.661
**Age + BMI + CTRP1**	0.860 (0.787–0.922)	98.51	0.509
**Age + BMI + WISP-1 + CTRP1**	0.940 (0.895–0.980)	70.94	0.720

Likelihood-ratio tests: age + BMI vs. age + BMI + WISP-1, χ^2^ = 43.64, *p* < 0.001; age + BMI vs. age + BMI + CTRP1, χ^2^ = 23.35, *p* < 0.001; and age + BMI + WISP-1 vs. full model, χ^2^ = 9.28, *p* = 0.002. AUC confidence intervals were estimated by bootstrap resampling; the optimism-corrected AUC of the full model was 0.926. **AUC**, area under the receiver operating characteristic curve; **AIC**, Akaike information criterion; **BMI**, body mass index; **CI**, confidence interval; **GDM**, gestational diabetes mellitus.

**Table 4:** Nested multivariable logistic regression models for GDM status.

Model/Variable	β	SE	OR	95% CI for OR	*p* Value
**Model 1: Maternal age + BMI**					
Intercept	−9.355	2.837	<0.001	<0.001–0.023	0.001
Maternal age, per year	0.022	0.050	1.02	0.93–1.13	0.659
BMI, per kg/m^2^	0.310	0.074	1.36	1.18–1.58	<0.001
**Model 2: Maternal age + BMI + WISP-1**					
Intercept	−6.169	3.753	0.002	<0.001–3.28	0.100
Maternal age, per year	−0.015	0.069	0.99	0.86–1.13	0.828
BMI, per kg/m^2^	0.244	0.103	1.28	1.04–1.56	0.018
WISP-1, per 1-SD increase	2.280	0.464	9.78	3.94–24.27	<0.001
**Model 3: Maternal age + BMI + WISP-1 + CTRP1**					
Intercept	−4.907	3.710	0.007	<0.001–10.63	0.186
Maternal age, per year	−0.043	0.074	0.96	0.83–1.11	0.562
BMI, per kg/m^2^	0.229	0.110	1.26	1.01–1.56	0.037
WISP-1, per 1-SD increase	2.019	0.478	7.53	2.95–19.21	<0.001
CTRP1, per 1-SD increase	1.283	0.466	3.61	1.45–8.99	0.006

**BMI**, body mass index; **CI**, confidence interval; **CTRP1**, C1q/TNF-related protein-1; **GDM**, gestational diabetes mellitus; **OR**, odds ratio; **SE**, standard error; **SD**, standard deviation; **WISP-1**, Wnt1-inducible signaling pathway protein-1.

## Data Availability

The datasets generated and/or analyzed in the current study are available from the corresponding author upon reasonable request. All data supporting the findings of this study are included within the article.

## Supplementary Materials

The following supporting information can be downloaded at https://www.mdpi.com/article/10.3390/medicina62101941/s1, Figure S1. Calibration of the final multivariable logistic regression model.
