# Supplementary material for: Serum WISP-1 and CTRP1 Levels in Gestational Diabetes Mellitus: Associations with HOMA-IR and Glycemic Status
Source: Medicina (Kaunas). 2026 Oct 8;62(10):1941. doi: 10.3390/medicina62101941 (PMC13707058; doi:10.3390/medicina62101941)

Supplementary Figure S1. Calibration of the final multivariable model

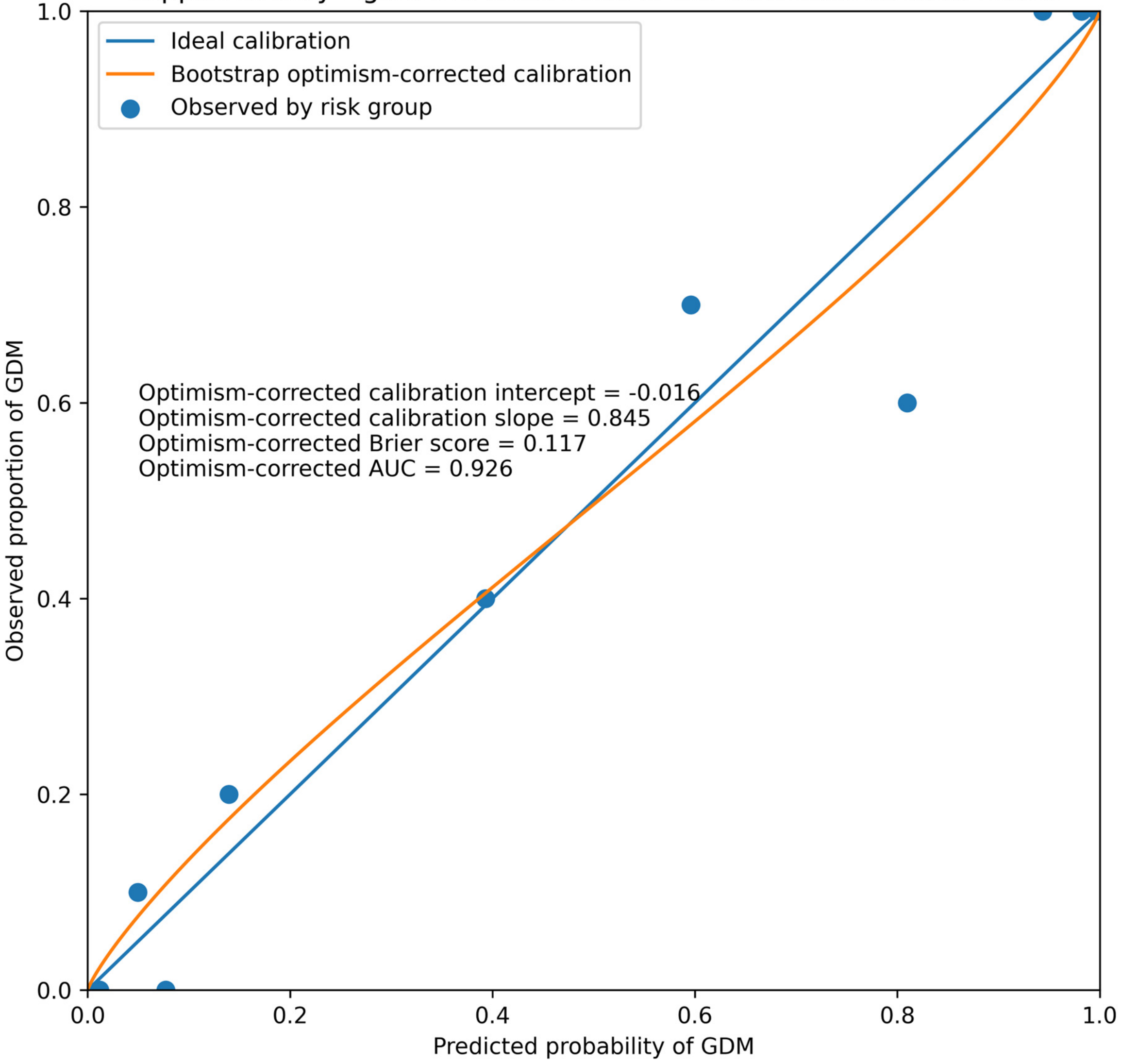

Supplement: Supplementary file 1 [file medicina-62-01941-s001.zip › medicina-4574030-supplementary.pdf]
